# Supplementary material for: Identification of TRPM2 as a Potential Therapeutic Target Associated with Immune Infiltration: A Comprehensive Pan-Cancer Analysis and Experimental Verification in Ovarian Cancer
Source: Int J Mol Sci. 2023 Jul 25;24(15):11912. doi: 10.3390/ijms241511912 (PMC10418504; doi:10.3390/ijms241511912)
Supplement: Supplementary file 1 [file ijms-24-11912-s001.zip › Figures S1-S4.pdf]

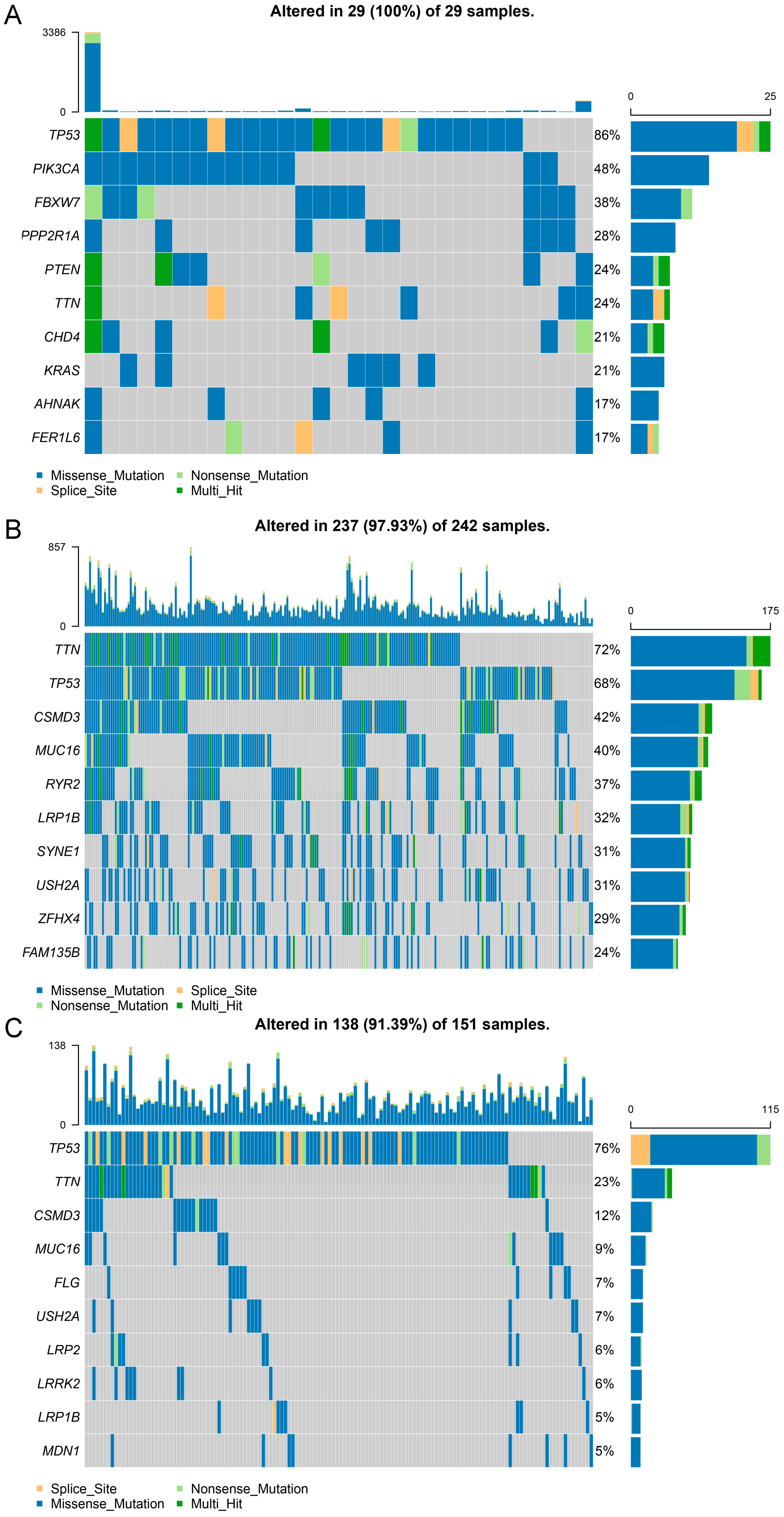

Figure S1. Waterfall showing the top mutated genes and mutated types in the high TRPM2 expression patients with UCS (**A**), OV (**B**), and LUSC (**C**).

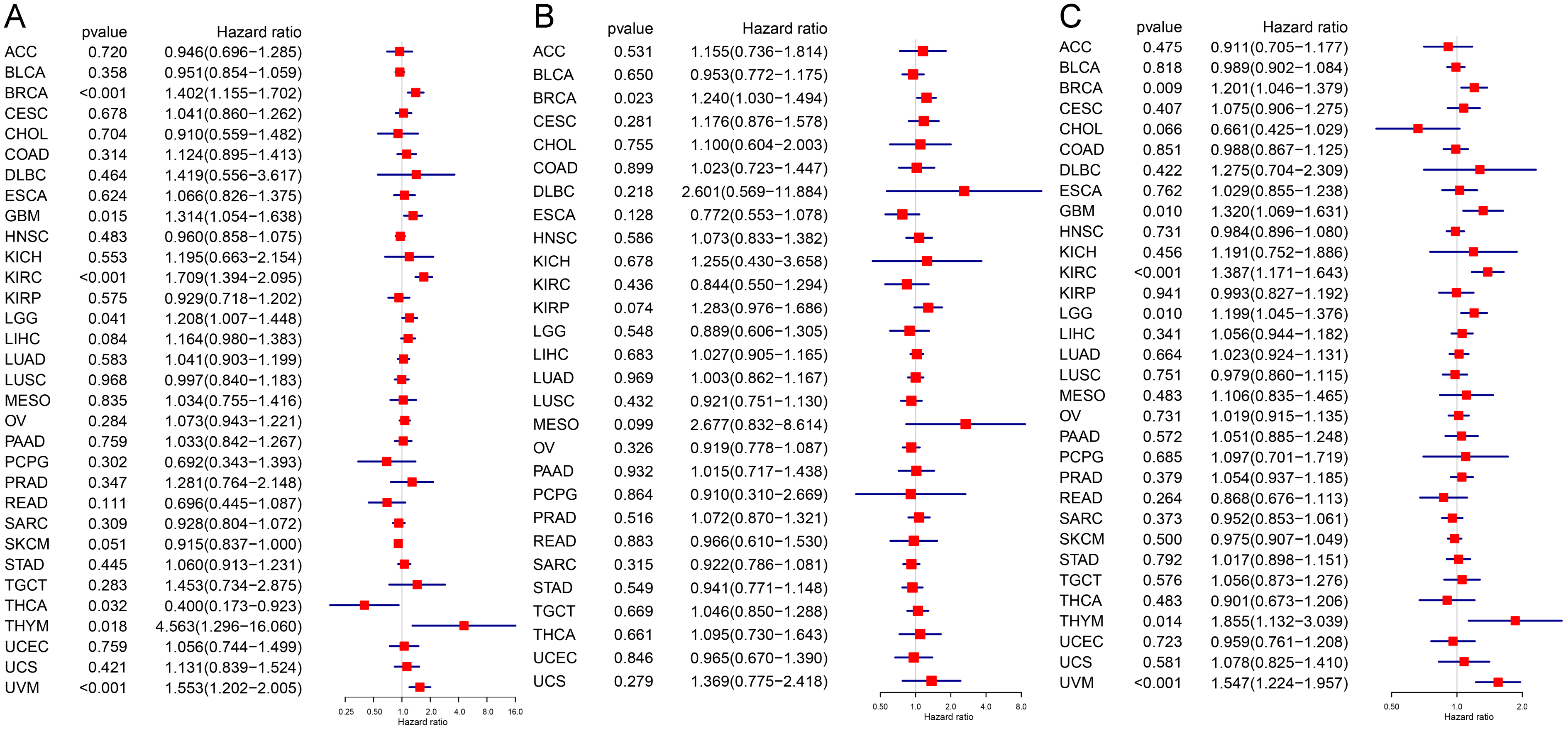

Figure S2. Univariate COX regression analysis of TRPM2 with (**A**) DSS; (**B**) DFI; (**C**) PFI.

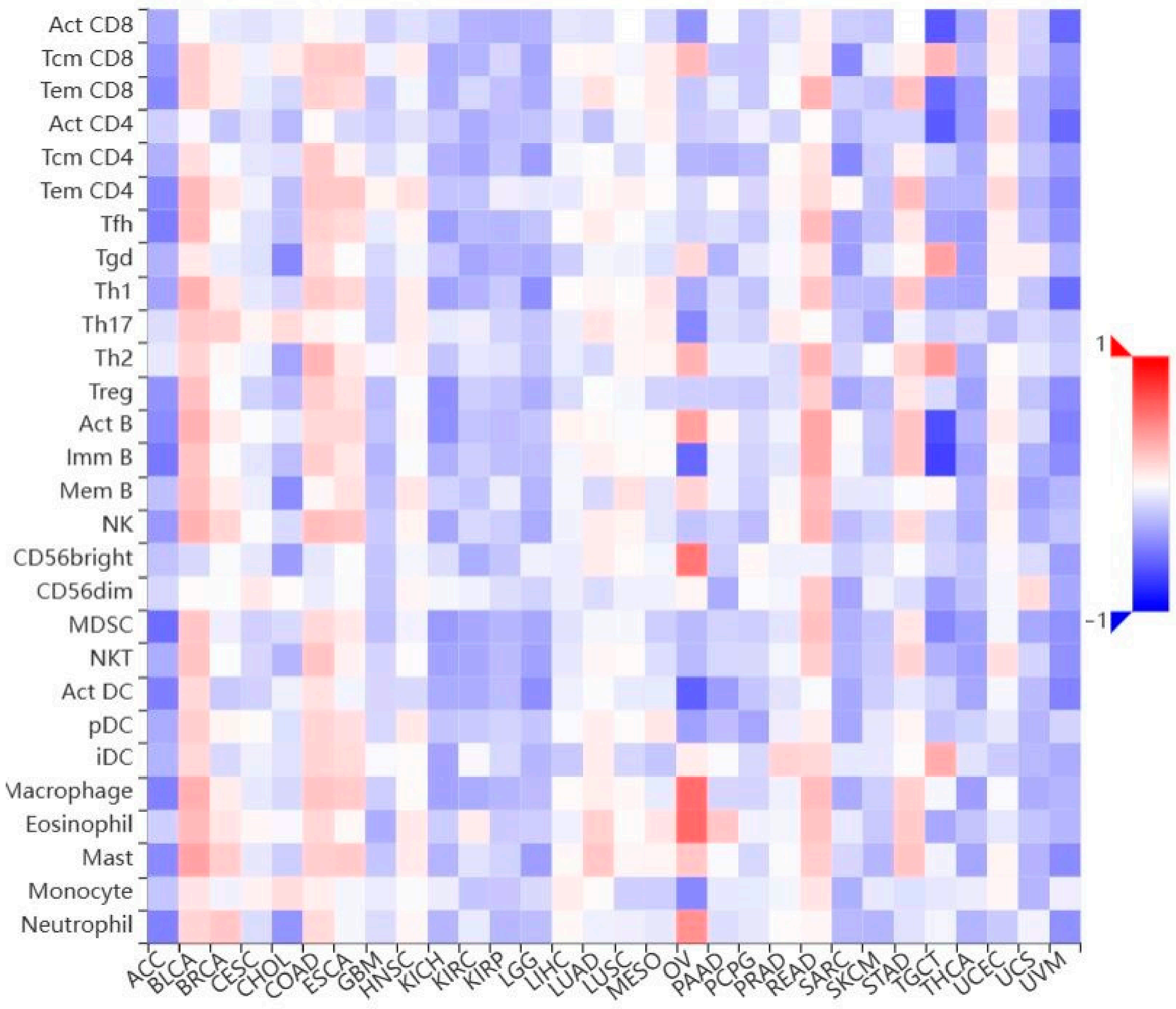

Figure S3. The correlation between methylation of TRPM2 and immunocyte infiltration.

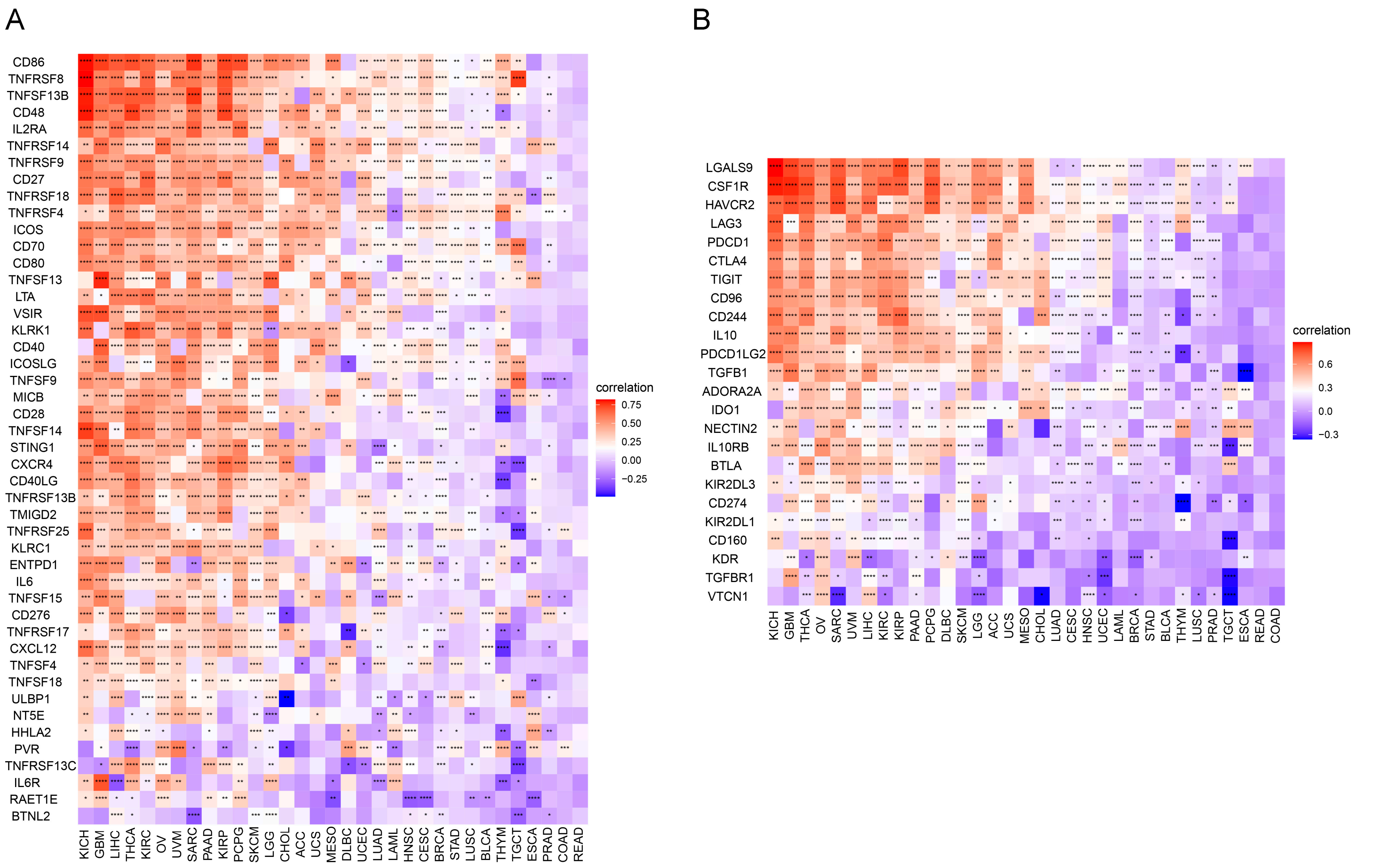

Figure S4. Correlation of TRPM2 expression with (**A**) immune activation genes; (**B**) immunosuppressive genes. * Indicates p-value < 0.05; ** p < 0.01; *** p < 0.001; **** p < 0.0001.
